# The Possibility of Acute Drug-Induced Liver Injury Associated With Prednisolone

**DOI:** 10.7759/cureus.54227

**Published:** 2024-02-15

**Authors:** Hiroshi Okano, Mikio Takagi, Katsumi Mukai, Akira Nishimura, Kana Asakawa, Youichirou Baba, Tetsuya Murata

**Affiliations:** 1 Gastroenterology, Suzuka General Hospital, Suzuka, JPN; 2 Nephrology, Suzuka General Hospital, Suzuka, JPN; 3 Pathology, Suzuka General Hospital, Suzuka, JPN

**Keywords:** corticosteroid-induced liver damage, drug-induced lymphocyte-stimulation test, low-dose corticosteroid therapy, prednisolone, drug-induced liver injury

## Abstract

A female patient was referred to our hospital with complaints of liver injury. She had been treated for immunoglobulin (Ig)A nephropathy using prednisolone and other medications. Drug-induced liver injury (DILI) was suspected, as no evidence of viral infection or autoimmune liver disease was apparent. All medications except for prednisolone were discontinued, but liver enzyme levels remained elevated. Percutaneous liver biopsy showed the characteristics of DILI and drug lymphocyte stimulation testing yielded positive results for prednisolone. After stopping administration of prednisolone, liver enzyme levels recovered to near-normal. Prednisolone has immunosuppressive effects and is sometimes used to treat DILI. Some reports have revealed that high-dose corticosteroids can induce liver injury, but liver injuries associated with low-dose corticosteroid therapy have not been described. Prednisolone-induced liver injury is a rare phenomenon. When low-dose corticosteroids are used for treatment, care should be taken regarding the possibility of liver injury.

## Introduction

The number of cases of drug-induced liver injury (DILI) has reportedly been increasing [1]. Acetaminophen, antiretroviral anti-infectious, psychoactive, and lipid-lowering agents, and non-steroidal anti-inflammatory drugs (NSAIDs) have been reported as associated with this pathology [1,2]. Traditional alternative medicines have been reported as the most common cause of DILI in Asia, according to the previous report [1]. In contrast to paracetamol-induced hepatotoxicity derived from dose-dependent overdose of the drug, most cases of DILI are due to idiosyncratic or unexpected reactions [3]. The mechanisms responsible for hepatocellular DILI are largely unknown [4]. Many different mechanisms may be involved in the hepatotoxicity of DILI, including immune reactions [5]. Corticosteroids were the first immunosuppressive agents to be used clinically [6] and remain one of the most important classes of immunosuppressive agents. The immunosuppressive characteristics of corticosteroids are sometimes used in the treatment of DILI [7-9]. While some cases of DILI resulting from corticosteroid use have been reported [10-13], this phenomenon is rare. In this case, we identified a female patient with acute DILI associated with oral prednisolone.

## Case presentation

**Figure 1:**
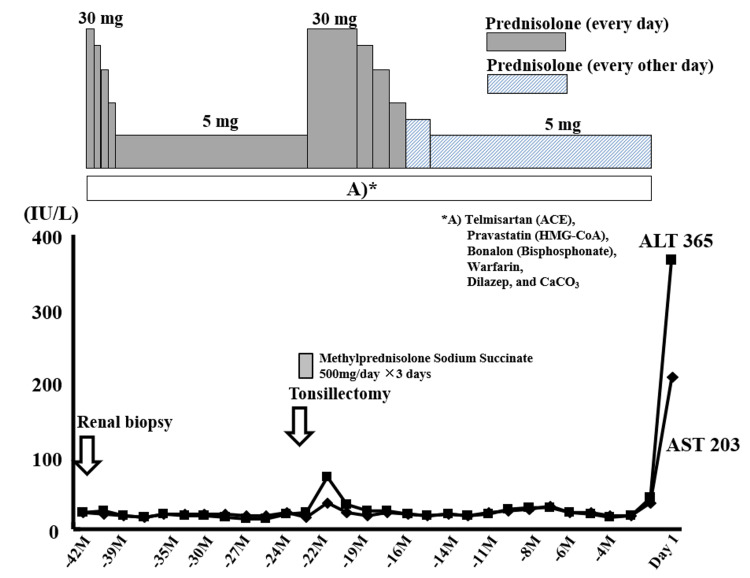
Clinical course before referral to the gastroenterological department. For a nephrotic syndrome derived from immunoglobulin A nephropathy, the patient had been administered low-dose oral prednisolone for a long period before the onset of liver injury. During follow-up, she was undergone tonsillectomy. The initial oral prednisolone dose for IgA nephropathy with nephrotic syndrome was 30 mg/day. This was subsequently tapered to either 5 mg/day for approximately three months (first introduction) or 5 mg every other day for approximately six months (after exacerbation). AST, aspartate aminotransferase; ALT, alanine aminotransferase.

A 40-year-old woman was referred to the gastroenterology department of our hospital due to liver injury. The patient was first identified with proteinuria at the age of 23. At 25, she was referred to our nephrology department due to suspected chronic nephritis diagnosed at another clinic. Although recommended to undergo a renal biopsy for diagnosis, she opted for outpatient follow-up without biopsy at her request. Her proteinuria remained stable at around 0.5-1.0g/day. At 36, upon persuasion from her medical attendant and given the persistent proteinuria, she underwent a percutaneous renal biopsy at another specialty facility. The biopsy confirmed IgA nephropathy. Oral prednisolone was started at 30mg/day (-42 months) and later tapered to 5mg/day (Figure 1). An exacerbation of IgA nephropathy with increased urinary protein excretion was identified at 38 years old (-22 months) (Figure 1). The patient underwent tonsillectomy, and the prednisolone dose, previously maintained at 5mg/day, was increased to 30mg/day following a three-day pulse therapy of methylprednisolone sodium succinate 500mg/day. During follow-up with prednisolone at 30mg/day, her liver enzymes transiently elevated but normalized spontaneously (Figure 1). The prednisolone was tapered to 5mg every other day, and her liver enzymes remained within normal limits until her recent episode of liver injury (day 1).

On presentation, the patient was asymptomatic. Her height was 153 cm and weight was 78.2 kg (body mass index, 33.4). The abdomen was soft and flat, without palpable masses or hepatomegaly. Splenomegaly was not detected. Consciousness was normal. Blood pressure was 128/96 mmHg, heart rate was 95 beats/min, and peripheral oxygen saturation was approximately 98% in room air. Body temperature was 35.8°C. She was taking oral prednisolone 5 mg every other day. She did not drink alcohol or smoke and had no history of blood transfusions or herbal remedy use.

**Figure 2:**
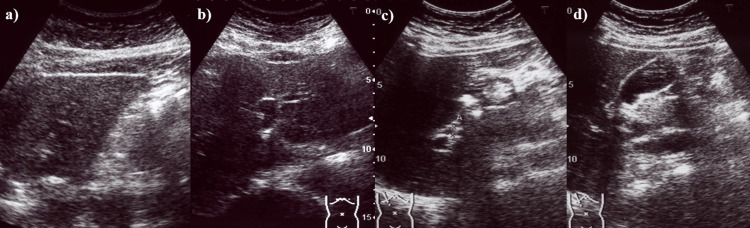
Abdominal ultrasonography (US) of the liver. (a) The liver had a dull edge. (b, c) No mass was detected in the liver, and no dilatations were seen in either of the intrahepatic bile ducts or in the common bile duct. (d) The gallbladder showed no abnormalities on imaging.

Laboratory data showed elevated levels of hepatic enzymes (Table 1), including aspartate aminotransferase (AST), alanine aminotransferase (ALT), and lactate dehydrogenase (LDH). Alkaline phosphatase (ALP) and total bilirubin (T-Bil) were within normal limits. Serum creatinine level was mildly elevated. Auto-antibodies, viral infection markers, and coagulation tests were normal. All immunoglobulin levels were within normal ranges. Urinalysis showed proteinuria (Table 1). Abdominal ultrasonography (US) revealed no significant findings except for a dull edge of the liver (Figures 2a-2d).

**Table 1:** Laboratory data on reference to the gastroenterology department. CBC, complete blood count; WBC, white blood cells; RBC, red blood cells; PT, prothrombin time; TP, total protein; Alb, albumin; AST, aspartate aminotransferase; ALT, alanine aminotransferase; LDH, lactate dehydrogenase; ALP, alkaline phosphatase; g-GT, g-glutamyltransferase; T-Bil, total bilirubin; ChE, Cholinesterase; T-Chol, total cholesterol; BUN, blood urea nitrogen; UA, uric acid; Crea, Creatinine; eGFR, estimated Glomerular Filtration Rate; HBsAg; hepatitis B surface antigen; COI, cut-off index; HCVAb, anti-hepatitis C virus antibody; IgG-HBc, Immunoglobulin G antibody to the hepatitis B core antigen; IgM-HSV, Immunoglobulin M antibody to herpes simplex virus; IgM-CMV, Immunoglobulin M antibody to cytomegalovirus; IgM-EBV, Immunoglobulin M antibody to Epstein-Barr virus; EBNA, Epstein-Barr virus-nuclear antibody; IgM-HAV, Immunoglobulin M antibody to hepatitis A virus; HEV RNA, hepatitis E virus ribonucleic acid; HCVRNA, hepatitis C virus ribonucleic acid; IgG, Immunoglobulin G; IgA, Immunoglobulin A; IgM, Immunoglobulin M; ANA, Anti-nuclear antibody; AMAM2, anti-mitochondrial antibody M2 subtype. #1: Serum ALP levels measured using the International Federation of Clinical Chemistry and Laboratory Medicine (IFCC) method could be calculated as 0.34 times the ALP levels measured using the Japan Society of Clinical Chemistry (JSCC) method.

	Data	Reference
CBC		
WBC (/mL)	7500	3900-9800
(Eosinophils)	(3.3%)	-
RBC (/mL)	462 × 10^4^	427-570
Hemoglobin (g/dL)	14.2	13.5-17.6
Hematocrit (%)	41.8	39.8-51.8
Platelets (/mL)	29.0×10^4^	130-369
Coagulation		
PT (%)	87	70-130
Chemistry		
TP (g/dL)	6.6	6.5-8.5
Alb (g/dL)	4.9	4.1-5.3
AST (IU/L)	266	10-35
ALT (IU/L)	472	10-35
LDH (IU/L) (IFCC)	405	124-222
ALP (IU/L) (IFCC)	61^#1^	72-113
g-GT (IU/L)	77	8-60
T-Bil (mg/dL)	0.6	0.2-1.3
ChE (IU/L)	380	229-520
T-Chol (mg/dL)	217	150-219
BUN (mg/dL)	15.0	9.6- 22.0
UA (mg/dL)	9.1	2.0-6.9
Crea (mg/dL)	1.18	0.50-1.10
eGFR (mL/min/1.73m^2^)	41.5	0.00-0.30
Viral markers		
HBsAg (COI)	(-)	-
HCVAb (COI)	(-)	-
IgG-HBc	(-)	-
IgM-HSV	(-)	-
IgM-CMV	(-)	-
IgM-EBV	(-)	-
EBNA	<10	0.0-9.9
IgM-HAV	(-)	-
HEV RNA	(-)	-
HCVRNA (logIU/mL)	(-)	0.0-1.1
Serology		
IgG (mg/dL)	844	870-1700
IgA (mg/dL)	190	110-410
IgM (mg/dL)	126	35-220
ANA	(-)	0-39
AMAM2	(-)	0.0-6.9

**Figure 3:**
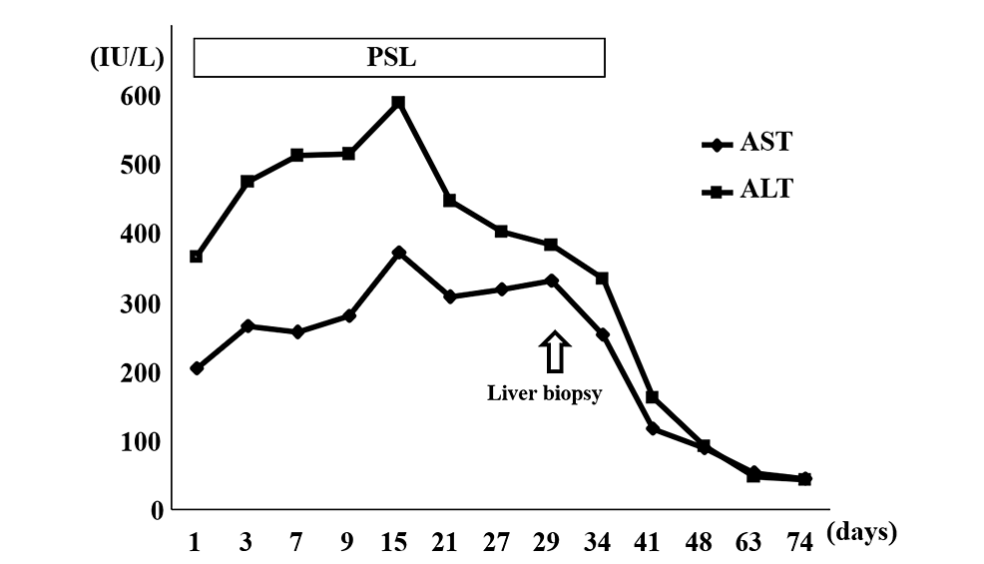
Clinical course after reference to the department of gastroenterology. Under continuing prednisolone administration, AST and ALT levels remained >200 IU/L. However, these values recovered lower than 50 IU/L after cessation of prednisolone.

**Figure 4:**
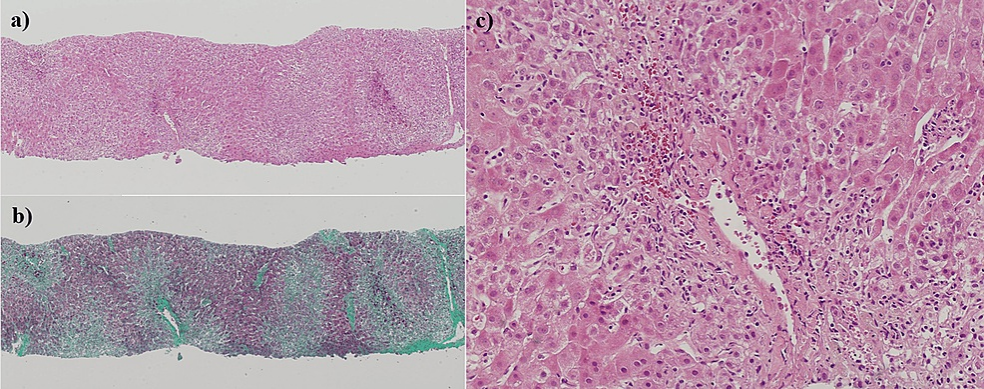
Histological findings of the liver. (a) Hematoxylin and eosin stain, magnification x10. (b) Azan stain, magnification x10. (c) Hematoxylin and eosin stain, magnification x40. Histopathological studies showed necrosis with hemorrhage in the centrilobular zone of the liver. Fatty changes of hepatocytes were not shown. These histopathological changes were thought to be a result of drug-induced liver injury.

At the time of referral, we suspected that one or more of the patient's medications were causing the liver injury. We initially considered stopping all medications to address the liver injury but decided against stopping prednisolone due to its importance in treating IgA nephropathy and its reported utility in some cases of DILI [3]. Although ALT levels improved after withdrawing the other suspect medications, they remained above 300 IU/L (Figure 3). AST levels continued to increase after drug cessation (Figure 3). After one month, the patient underwent an abdominal US-guided liver biopsy for differential diagnosis of the liver injury. Histopathological examination of the biopsy specimens showed centrilobular necrosis with hemorrhage (Figures 4a-4c). Fatty change of the liver was not apparent in the specimen. Additionally, a drug-induced lymphocyte-stimulation test (DLST) with prednisolone yielded positive results (Table 2). Based on these findings, liver injury was attributed to prednisolone use. Prednisolone was therefore discontinued, and AST and ALT levels returned to near-normal within one month (Figure 3). The value of T-Bil remained within normal limits during follow-up (data not shown). Due to the concern for prednisolone-induced hepatitis, all other medications except prednisolone were restarted after liver enzyme levels normalized. Despite this, the hepatitis did not recur. Therefore, we ultimately diagnosed the patient with prednisolone-induced DILI.

**Table 2:** Result of drug lymphocyte stimulation test. cpm, count per minute.

Drug	Measured value (cpm)	Stimulation index (%)	Judge
Prednisolone	386	244	(+)
Control	158	-	

Considering the cessation of prednisolone, the patient is currently receiving mizoribine 100mg/day. While her liver enzyme levels remain within normal range, a switch to alternative medications may be necessary due to the estimated daily urine protein excretion of 1-2 grams and an eGFR of 30-40.

## Discussion

DILI is a major concern for both patients and healthcare providers [4]. Drugs are estimated to cause about 10% of all fulminant hepatitis cases in Japan and 15% to 20% of cases in Western countries [14,15]. Because no specific markers or tests are generally available for DILI diagnosis, early detection during acute liver injury can be difficult [14]. Therefore, a prudent differential diagnosis is crucial. For suspected DILI cases, careful consideration of recent comprehensive reports on the disease is essential [1], and causality assessments must rely on chronological and clinical criteria [14]. For example, the DDW-J scale is a powerful tool with potentially valuable clinical applications in DILI diagnosis [16]. However, many cases remain unidentified, with estimates suggesting that only around 10% are ultimately diagnosed [14].

Several side effects of corticosteroid therapy are well-known, particularly steatohepatitis [17,18]. However, despite histopathological examination of liver biopsy specimens, we did not observe steatohepatitis in this case. Therefore, we believe that the patient's liver injury was not related to any abnormalities in fat metabolism resulting from long-term, low-dose prednisolone administration.

A limited number of reports have described corticosteroid-induced liver damage, primarily following high-dose methylprednisolone therapy [10-13]. However, the present case developed liver injury during maintenance therapy with low-dose prednisolone (5 mg every other day). A review of the English literature revealed no published reports of liver injury induced by low-dose oral steroids. This case may represent the first documented instance of acute DILI associated with low-dose oral prednisolone. Although the high-dose prednisolone administration approximately two years prior to hepatitis onset may be relevant to the development of acute hepatitis, the mechanisms underlying this association remain unclear. While Ueno et al. reported a link between drug-induced autoimmune-like hepatitis and hepatitis following high-dose methylprednisolone treatment, no evidence of autoimmune hepatitis was found in this case.

Generally, DILI would be expected to improve after discontinuing the causative drug. However, due to the importance of various pharmacotherapies in most treatment regimens, the decision to suspend suspected agents should be carefully considered. In clinical practice, physicians should exercise caution when diagnosing DILI and identifying causal drugs, as permanently discontinuing a medication could worsen the underlying disease being treated.

In this case, the liver injury occurred approximately 22 months after the increase in prednisolone dosage. Possible causes include contamination of the medication or the use of an undisclosed herbal or natural supplement. However, the rapid decline in liver enzyme levels following prednisolone discontinuation suggests a potential association between the medication and liver injury.

## Conclusions

We diagnosed the present patient with suspected DILI caused by prednisolone. This case highlights the importance of considering corticosteroids as a potential cause of DILI, even though they exert immunosuppressive effects and are sometimes used to treat DILI itself. The available literature suggests that high-dose corticosteroid therapy is a potential risk factor for DILI. However, low-dose, long-term corticosteroid use may also represent another risk factor for DILI. Corticosteroid therapy is now employed for a wide range of disease categories and is commonly prescribed. Therefore, the possibility of DILI induced by prednisolone should be considered in clinical practice.
